# Venetoclax and Blinatumomab for adult patients with relapsed/refractory or MRD positive Ph-negative B-cell precursor ALL: phase I part of the GMALL-BLIVEN trial

**DOI:** 10.1007/s00277-026-06883-8

**Published:** 2026-02-12

**Authors:** Lars Fransecky, Walter Fiedler, Christoph Röllig, Tim Sauer, Simon Raffel, Winfried Alsdorf, Lisa Heberling, Britta Steer, Karin Huber, Tim Versteegen, Lea Spory, Dorothee Deckbar, Lena Reiser, Sonja Bendig, Lorenz Bastian, Cecilia Bozzetti, Thomas Beder, Martin Neumann, Monika Brüggemann, Nicola Gökbuget, Claudia Baldus

**Affiliations:** 1 Department of Internal Medicine II (Hematology/Oncology), University Hospital Schleswig-Holstein, Campus Kiel, Kiel, Germany; 2 University Cancer Center Schleswig-Holstein (UCCSH), University Hospital Schleswig-Holstein, Kiel, Germany; 3 Department of Oncology and Hematology with Section Pneumology, University Medical Center Hamburg-Eppendorf, University Cancer Center Hamburg, Hamburg, Germany; 4 Department of Internal Medicine I, University Hospital TU Dresden, Dresden, Germany; 5 Department of Internal Medicine V, Heidelberg University Hospital, Heidelberg, Germany; 6 Clinical Research Unit “CATCH ALL” (KFO 5010) funded by the Deutsche Forschungsgemeinschaft, Bonn, Germany; 7 Department of Internal Medicine II, Hematology and Oncology, Goethe University Hospital Frankfurt, Frankfurt, Germany

**Keywords:** Acute leukemia, Acute lymphoblastic leukemia (ALL), Venetoclax, Blinatumomab, Bispecific antibody, BH3 mimetics, Measurable residual disease

## Abstract

Prognosis of adult patients with relapsed/refractory (r/r) or measurable residual disease (MRD)–positive B-precursor acute lymphoblastic leukemia (B-ALL) remains poor. Blinatumomab induces complete remissions (CR) and MRD negativity in a subset of patients, yet overall survival remains limited. As BCL2 overexpression contributes to leukemic cell survival and therapy resistance, combining Blinatumomab with the BCL2 inhibitor Venetoclax may enhance therapeutic efficacy. The GMALL-BLIVEN (NCT05182385) phase I multicenter trial evaluated the safety and feasibility of Venetoclax plus Blinatumomab in adults with CD19⁺, Philadelphia chromosome–negative r/r or MRD-positive B-ALL. Dose escalation followed a 3 + 3 design across three Venetoclax dose levels (DL-1: 400 mg; DL-2: 600 mg; DL-3: 800 mg) administered from day − 7 to day 42, with Blinatumomab given per label. The primary endpoint was determination of the maximum tolerated dose (MTD); key secondary endpoint was achievement of molecular complete remission (MOL-CR) by centralized IG/TR MRD assessment (sensitivity ≥ 10⁻⁴). Nine patients (median age 52 years, range 22–71) were enrolled across four German centers: four with r/r B-ALL and five with MRD-positive disease. Molecular subtypes included ZNF384-rearranged (n = 2), BCR::ABL1-like (n = 2), CEBP-rearranged (n = 1), hypodiploidy (n = 1), B-ALL NOS (n = 2), and KMT2A-rearranged (n = 1). No dose-limiting toxicities were observed, and the MTD was not reached. Treatment-emergent grade ≥ III toxicities included neutropenia, febrile neutropenia, cytokine release syndrome, and ICANS; no 30- or 60-day mortality occurred. Six patients completed two full cycles without treatment interruptions. The recommended phase II dose (RP2D) was established as Venetoclax 800 mg daily plus standard-dose Blinatumomab. All nine patients were evaluable for response. Among r/r B-ALL, responses included PR (*n* = 1), CR (*n* = 1), and PD (*n* = 2). Among MRD-positive patients, 4/5 achieved MOL-CR. Four patients were successfully bridged to allogeneic stem cell transplantation. After a median follow-up of 17.8 months, median overall survival was 15.0 months (r/r: 6.6 months; MRD-positive: 16.9 months). Non-responders were enriched for adverse molecular subtypes (BCR::ABL1-like and hypodiploid B-ALL). Venetoclax combined with Blinatumomab is safe and feasible in adults with r/r or MRD-positive B-ALL, without increased rates of CRS or neurotoxicity compared with Blinatumomab alone. High rates of MRD clearance were observed in MRD-positive patients. The phase II portion of GMALL-BLIVEN is ongoing at the RP2D of Venetoclax 800 mg daily.

**Supplementary Information:**

The online version contains supplementary material available at 10.1007/s00277-026-06883-8.

## Key Points

- It was safe to combine Venetoclax with Blinatumomab in r/r B-ALL
- Recommended Phase-II-Dose was defined as continuous Venetoclax 800 mg/d in combination with standard dose Blinatumomab

**Supplementary Information:**

The online version contains supplementary material available at 10.1007/s00277-026-06883-8.

Prognosis of adult patients with relapsed/refractory (r/r) B-precursor acute lymphoblastic leukemia (B-ALL) remains poor. Monotherapy with Blinatumomab in r/r B-ALL induced complete remissions (CR) in 44% of patients and measurable residual disease (MRD) negative molecular remissions (MOL-CR) in 33% with a median overall survival (OS) of only 7.7 months [1]. In adult patients with MRD positive B-ALL, Blinatumomab monotherapy led to MOL-CR in 78% of patients and to a median OS of 36.5 months with subsequent allogeneic stem cell transplantation (SCT) performed in 70% of the patients [2]. Although Blinatumomab monotherapy has a favorable safety profile, its clinical efficacy depends on tumor burden at relapse; therefore, combinations with other treatments may enhance effectiveness.

BCL2 is a critical regulator of the intrinsic apoptotic pathway and is frequently overexpressed in B-ALL. By sequestering proapoptotic factors such as BIM and BAD, BCL2 promotes leukemic cell survival and confers resistance to conventional cytotoxic therapies [3, 4]. Overexpression of BCL2 in B-ALL has been linked to poor prognosis, as malignant blasts may evade apoptosis even in the presence of genotoxic stress [5, 6]. Consequently, this antiapoptotic protein has emerged as an attractive therapeutic target: disrupting its function can restore the normal apoptotic cascade and sensitize leukemic cells to both chemotherapy and immunotherapy [7, 8]. Small-molecule inhibitors, like Venetoclax that specifically block BCL2 offer a promising avenue to enhance treatment efficacy, particularly when combined with other targeted agents or immunotherapies [9, 10]. Preclinical and clinical investigations suggest activity of Venetoclax in B-ALL [11–14].

The GMALL study group developed this phase 1 multicenter clinical trial to evaluate the safety of Blinatumomab in combination with Venetoclax in r/r and MRD positive B-ALL patients (GMALL-BLIVEN, NCT05182385). The trial was conducted in accordance with the principles of Good Clinical Practice (GCP) guidelines and the Declaration of Helsinki, and approved by the ethics committees of the participating sites. GMALL-BLIVEN included adult patients with CD19-positive Ph-negative B-ALL (aged 18 years and older with no upper age limit) after written informed consent prior to trial partizipation. Patients had either r/r B-ALL (bone marrow blasts ≥ 5%) or MRD positive B-ALL (MOL-failure/MOL-relapse, i.e. bone marrow blasts < 5%, and MRD >1xE-04). To assess safety in this exploratory study with a small sample size, we determined the maximum tolerated dose (MTD) as a primary endpoint of the phase I part of the trial using a 3 + 3 design, where three patients will form a cohort. Each cohort will receive a higher cumulative dose level (DL) of Venetoclax ranging from 400 mg/d (DL-1) to 800 mg/d (DL-3). If one patient experiences dose limiting toxicity (DLT), the cohort will be expanded to six patients. If two or more of these six patients experience a DLT, the next lower Venetoclax dose will be defined as maximum tolerated dose (MTD). If 0/3 or < 2/6 patients in a cohort experience a DLT, the next dose escalation cohort will be opened. We defined DLT evaluation period as the first 49 days after initiation of Venetoclax in cycle 1. Key secondary endpoint was a MOL-CR, defined as MRD negativity measured by real-time quantitative PCR (RQ-PCR) of clonal immunoglobulin and T-cell receptor (IG/TR) rearrangements with a minimum assay sensitivity of 1xE-04 in the GMALL reference laboratory (Hämatologie Labor Kiel, Germany). Treatment consisted of once-daily oral Venetoclax on day − 7 to day 42 at three dose levels (DL-1 400 mg, DL-2 600 mg, DL-3 800 mg), and Blinatumomab according to the European Medicines Agency label, i.e. day 1–28 as continuous intravenous infusion of 28 µg/d 4 weeks on and 2 weeks off. In case of r/r B-ALL, a cautionary dose step of 9 µg/d Blinatumomab was implemented for one week.

A total of nine patients with a median age of 52.1 years (range 22–71 years) were enrolled in four German centers in this phase I part of GMALL-BLIVEN from February 2022 to May 2023. Median initial white blood cell count (WBC) was 12.5/nl (range 3.7–356.2/nl), including three patients with WBC > 30/nl at initial diagnosis, indicating high risk disease. Median time from initial diagnosis to study entry was 6.1 months (range 4.0–38.4 months). The target doses were 400 mg (*n* = 3, DL-1), 600 mg (*n* = 3, DL-2), and 800 mg (*n* = 3, DL-3) Venetoclax (Table 1). Central IG/TR-based MRD assessments were performed in all patients. Enrollment included patients with hematological relapse (*n* = 4), while five patients had MRD positive B-ALL. Among patients with r/r B-ALL, three of four patients had refractory disease during front-line therapy with intensive GMALL induction/consolidation therapy, while one patient suffered relapse shortly after failure of second allogeneic SCT.

**Table 1:** Patient characteristics at screening in the phase I part of the GMALL-BLIVEN trial. Molecular subtypes refer to the international consensus Classification, IG/TR level = abundance of IG/TR rearrangements as assessed by RQ-PCR,, Ph-like = *BCR::ABL1*-like, LH = low hypodiploid, NOS = not otherwise specified, GMALL int = treatment according to intensive, pediatric inspired GMALL study protocol, BLINA = Blinatumomab, r/r = relapsed/refractory, MRD = molecular relapse

Study ID	Age At Study Inclusion (Years)	Molecular Subtype	WBC at Initial Diagnosis (/nl)	Prior Therapy Lines	Time from Diagnosis to Inclusion (Months)	Relapse Type	BM Blasts (%)	IG/TR Level	Dose Level Ven (mg)
C1P03	32	BCR::ABL1-like	323.9	GMALL int	11.8	r/r	54	4.00E-01	400
C2P05	22	BCR::ABL1-like	72	GMALL int	31.1	r/r	85	5.00E-01	600
C3P07	71	NOS	6.1	GMALL int	4	r/r	5	1.00E-02	800
C3P09	68	LH	4.2	GMALL int	6.3	r/r	13	2.00E-01	800
C1P01	49	rZNF384	12.5	GMALL int	4.8	MRD	0	1.00E-03	400
C1P02	40	KMT2A-r	356.2	GMALL int	5.1	MRD	0	4.00E-03	400
C2P04	70	rZNF384	15.3	GMALL int, FLAG-IDA	29.7	MRD	3	1.00E-03	600
C2P06	58	NOS	3.7	GMALL int	4.2	MRD	2	4.00E-04	600
C3P08	59	rCEBP	7.9	GMALL int, BLINA	38.4	MRD	2	1.00E-04	800

**Fig. 1:**
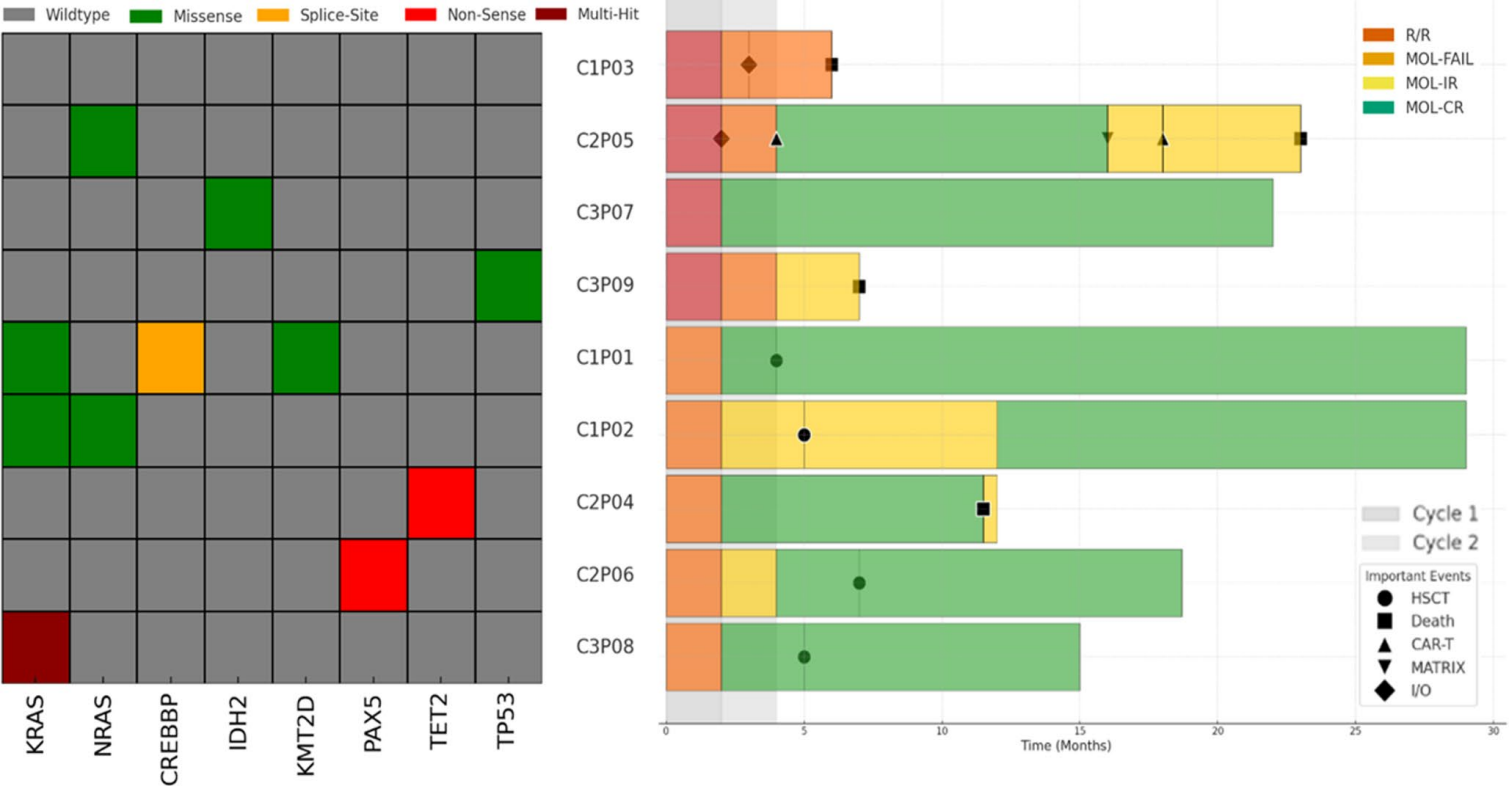
Clinical and molecular data of *n* = 9 patients treated within the Phase I part of the GMALL-BLIVEN trial. Left panel: mutational landscape. Each row represents a patient, each column represents a gene/alteration. Right panel: Swimlane plot indicating individual patient outcomes. Important events, such as subsequent therapies and clinical outcomes are labelled. CAR-T, chimeric antigen receptor T-cell therapy, HSCT, allogeneic hematopoietic stem cell transplantation, MATRIX, intensive chemotherapy protocol including Methotrexate, Cytarabine, Thiotepa, and Rituximab. I/O, Inotuzumab-Ozogamycin, MOL-IR: detectable MRD below the quantitative threshold; MOL-CR: no detectable MRD in CR; MOL-FAIL: failure to achieve MOL-CR or MOL-IR

Molecular B-ALL subtypes were identified by transcriptome [15] and panel DNA sequencing in all nine analyzed patients, including *ZNF384*-rearranged (*n* = 2), *BCR::ABL1*-like (*n* = 2; *IGH::CRLF2* rearranged), *CEBP*-rearranged (*n* = 1), Hypodiploidy (*n* = 1), B-ALL, not otherwise specified (NOS, *n* = 2) and *KMT2A*-rearranged B-ALL (*n* = 1). Mutation analysis revealed mutations typical for a cohort of B-ALL (Fig. 1), including mutations in *TP53* (*n* = 1), *PAX5* (*n* = 1*)*, and interestingly *IDH2* (*n* = 1).

Treatment-emergent serious adverse events (SAE) occurred in five of nine patients, including SAE of special interest, such as neutropenia (*n* = 1, CTCAE III°), febrile neutropenia (*n* = 1, CTCAE III°), Cytokine Release Syndrome (CRS, *n* = 2, CTCAE II°), Immune effector cell-associated Neurotoxicity Syndrome (ICANS, *n* = 2, ASTCT III°) and central line occlusion (*n* = 1, CTCAE II°) (Supplemental Table 1). Neutropenia was classified as severe adverse reaction (SAR) related to Venetoclax and resolved with G-CSF support before dose limiting toxicities (DLT) evaluation time elapsed. Six of nine patients received two full treatment cycles of BLIVEN, and no treatment interruption was associated with toxicity. Neither 30- nor 60-day mortalities after treatment with Blinatumomab and Venetoclax were observed.

Based on the evaluation of these safety data by an independent Data Safety Monitoring Board, no DLT were identified and the maximum tolerated dose was not reached. Thus, we defined the highest tested dose level (DL-3) with a daily dose of 800 mg Venetoclax in combination with standard dose Blinatumomab as recommended phase II dose (RP2D).

Response assessments were available for all patients. We found no correlation between dose level and response. Treatment with Blinatumomab/Venetoclax of four patients with r/r B-ALL resulted in PR (*n* = 1), CR (*n* = 1), and PD (*n* = 2). Of five patients enrolled in MRD positive B-ALL, four achieved a MOL-CR. After a median follow-up of 17.8 months (range: 6.1–29.1 months), four patients were successfully bridged to allogeneic SCT. Two patients achieving complete molecular remission did not proceed to allogeneic SCT due to advanced patient age (≥ 70years). There were four deaths after completion of the study during follow-up, which were all due to progressive disease (Fig. 1). Median overall survival for all patients, r/r B-ALL, and MRD positive B-ALL was 15.0 months, 6.6 months, and 16.9 months, respectively.

We concluded that combinational therapy with Blinatumomab and Venetoclax in adult patients with r/r and MRD positive B-ALL was safe and feasible. No treatment withdrawals were attributed to safety concerns. Most adverse events were compatible with underlying disease. Episodes of low-grade CRS and ICANS were noted in two cases, indicative of T-cell expansion and, importantly, without evidence of increased frequency compared to monotherapy with Blinatumomab.

Three study subjects did not respond to BLIVEN combination therapy. Of these, two patients in the cohort of r/r B-ALL exhibited *IGH::CRLF2* fusion transcripts defining a *BCR::ABL1* like B-ALL, while one r/r B-ALL patient had a low hypodiploidy B-ALL. Interestingly, the only CR in patients with r/r B-ALL was achieved in a 71-year-old patient harboring the *IDH2* R140Q hotspot mutation. *IDH2* R140Q mutations in B-ALL have been recently described in the context of prior treatment with immunomodulating agents for Multiple Myeloma [16]. Speculatively, B-ALL with *IDH2* mutations may be particularly sensitive to BCL2 inhibition as observed in *IDH2* mutated Acute Myeloid Leukemia [17]. All other patients included to this clinical trial were converted to MOL-CR, including five complete MRD responses, of which 2/2 occurred in *ZNF384* rearranged B-ALL.

In previous clinical trials, allogeneic SCT after Blinatumomab monotherapy had been performed in 24% and 66% in r/r and MRD positive B-ALL [1, 2], which was similar to our observed rate of allogeneic SCT of 0% and 80%. Median overall survival of 6.6 months and 16.9 months for r/r and MRD positive B-ALL was numerically shorter within this Phase I study of BLIVEN, when compared to the reported median survival times of 7.7 months and 36.5 months [1, 2]. However, evaluation of the therapeutic efficacy is limited by the low number of patients and the Phase I nature of this clinical trial. Further follow-up and larger patient numbers of the ongoing Phase II of the study will help to establish the potential benefit of this combination therapy. We anticipate that the role of this combination will be shaped by newer developments in Blinatumumab, particularly regarding improvements in its route of administration (e.g. subcutaneous delivery) and it´s expanding indications in the MRD negative setting.

In conclusion, it is safe to combine Venetoclax and Blinatumomab in adult patients with r/r B-ALL and MRD positive B-ALL. Enrollment into the phase II part of the GMALL-BLIVEN trial is currently ongoing at Venetoclax RP2D of 800 mg/d in combination with standard dose Blinatumomab.

## Supplementary Information

Below is the link to the electronic supplementary material.

Supplementary Material 1

## Data Availability

Data that support the findings of this study are available upon request.
