# Supplementary material for: Venetoclax and Blinatumomab for adult patients with relapsed/refractory or MRD positive Ph-negative B-cell precursor ALL: phase I part of the GMALL-BLIVEN trial
Source: Ann Hematol. 2026 Feb 12;105(3):109. doi: 10.1007/s00277-026-06883-8 (PMC12894111; doi:10.1007/s00277-026-06883-8)
Supplement: Supplementary file 1 — Supplementary Material 1 [file 277_2026_6883_MOESM1_ESM.docx]

**Supplemental Table 1**

Supplemental Table 1: Safety of nine patients enrolled to the phase I part of the GMALL-BLIVEN trial. Frequency of Adverse Events of special interest with severity grading according to CTCAE v5 and investigator-assessed relationship to study drugs Venetoclax or Blinatumomab. List includes all hematologic and non-hematologic adverse events reported by the investigators. ICANS=Immune Cell-associated Neurotoxicity Syndrome

| **Adverse Event** | **Any Grade** | **Grade ≥ 3** | **Severe Adverse Reaction** | **Association to Study drug** |
| --- | --- | --- | --- | --- |
| Neutropenia | 1/9 | 1/9 | yes | Venetoclax |
| Febrile Neutropenia | 1/9 | 1/9 | no | none |
| Sepsis | 0/9 | 0/9 | no | none |
| Invasive Aspergillosis | 0/9 | 0/9 | no | None |
| Hemorrhage | 0/9 | 0/9 | no | none |
| Cytokine Release Syndrome | 2/9 | 0/9 | yes | Blinatumomab |
| ICANS | 2/9 | 2/9 | yes | Blinatumomab |
| Central Line Occlusion | 1/9 | 0/9 | no | none |
